# AlphaFold Protein Structure Database: massively expanding the structural coverage of protein-sequence space with high-accuracy models

**DOI:** 10.1093/nar/gkab1061

**Published:** 2021-11-17

**Authors:** Mihaly Varadi, Stephen Anyango, Mandar Deshpande, Sreenath Nair, Cindy Natassia, Galabina Yordanova, David Yuan, Oana Stroe, Gemma Wood, Agata Laydon, Augustin Žídek, Tim Green, Kathryn Tunyasuvunakool, Stig Petersen, John Jumper, Ellen Clancy, Richard Green, Ankur Vora, Mira Lutfi, Michael Figurnov, Andrew Cowie, Nicole Hobbs, Pushmeet Kohli, Gerard Kleywegt, Ewan Birney, Demis Hassabis, Sameer Velankar

**Affiliations:** European Molecular Biology Laboratory, European Bioinformatics Institute, Hinxton, UK; European Molecular Biology Laboratory, European Bioinformatics Institute, Hinxton, UK; European Molecular Biology Laboratory, European Bioinformatics Institute, Hinxton, UK; European Molecular Biology Laboratory, European Bioinformatics Institute, Hinxton, UK; European Molecular Biology Laboratory, European Bioinformatics Institute, Hinxton, UK; European Molecular Biology Laboratory, European Bioinformatics Institute, Hinxton, UK; European Molecular Biology Laboratory, European Bioinformatics Institute, Hinxton, UK; European Molecular Biology Laboratory, European Bioinformatics Institute, Hinxton, UK; European Molecular Biology Laboratory, European Bioinformatics Institute, Hinxton, UK; DeepMind, London, UK; DeepMind, London, UK; DeepMind, London, UK; DeepMind, London, UK; DeepMind, London, UK; DeepMind, London, UK; DeepMind, London, UK; DeepMind, London, UK; DeepMind, London, UK; DeepMind, London, UK; DeepMind, London, UK; DeepMind, London, UK; DeepMind, London, UK; DeepMind, London, UK; European Molecular Biology Laboratory, European Bioinformatics Institute, Hinxton, UK; European Molecular Biology Laboratory, European Bioinformatics Institute, Hinxton, UK; DeepMind, London, UK; European Molecular Biology Laboratory, European Bioinformatics Institute, Hinxton, UK

## Abstract

The AlphaFold Protein Structure Database (AlphaFold DB, https://alphafold.ebi.ac.uk) is an openly accessible, extensive database of high-accuracy protein-structure predictions. Powered by AlphaFold v2.0 of DeepMind, it has enabled an unprecedented expansion of the structural coverage of the known protein-sequence space. AlphaFold DB provides programmatic access to and interactive visualization of predicted atomic coordinates, per-residue and pairwise model-confidence estimates and predicted aligned errors. The initial release of AlphaFold DB contains over 360,000 predicted structures across 21 model-organism proteomes, which will soon be expanded to cover most of the (over 100 million) representative sequences from the UniRef90 data set.

## Lay Summary

The AlphaFold Protein Structure Database (AlphaFold DB, https://alphafold.ebi.ac.uk) is an extensive, public database of highly accurate protein structure models. The models are the products of AlphaFold2, an Artificial Intelligence algorithm developed by DeepMind. AlphaFold enabled scientists to investigate an unprecedented number of protein structures. The database we describe here provides access to these predicted models and information on their accuracy. The first version of AlphaFold DB contains over 360,000 models of 21 biologically essential species.

## INTRODUCTION

Proteins are essential macromolecules with vital biological functions and, thus, are involved in a wide range of research activities and medical and biotechnological applications, from fighting infectious diseases to tackling environmental pollution (1,2). Knowledge of the three-dimensional (3D) arrangement of the atoms of a protein can provide essential clues to understanding the roles and mechanisms underpinning protein functions (3,4). However, while the Universal Protein Resource (UniProt) archives almost 220 million unique protein sequences, the Protein Data Bank (PDB) holds only just over 180 000 3D structures for over 55 000 distinct proteins, thus severely limiting the coverage of the sequence space to support biomolecular research globally (5–7).

Achieving a higher coverage of the sequence space with experimentally determined high-resolution structures is very labour-intensive. It often requires a lot of trial and error, for example, to find suitable constructs or conditions under which a protein is amenable to crystallization. Although recent advances in the field of electron cryo-microscopy and hybrid and integrative methods (I/HM) for structure determination have accelerated the pace of structure determination, the gap between known protein sequences and experimental protein structures continues to expand (6,8).

One way to close this gap is to predict the structures of millions of proteins. Increasingly, researchers deploy Artificial Intelligence (AI) techniques to predict a protein's structure computationally from its amino-acid sequence alone (9–11).

AlphaFold is an AI system developed by DeepMind that makes state-of-the-art predictions of protein structures from their amino-acid sequences (9). CASP (Critical Assessment of Structure Predictions) is a biennial challenge for research groups to test the accuracy of their predictions against actual experimental data. In 2020, the organizers of the CASP14 benchmark recognized AlphaFold as a solution to the protein–structure–prediction problem (12). The unprecedented accuracy and speed of AlphaFold allowed the creation of an extensive database of structure predictions at a large scale. It will enable biologists to obtain structural models for almost any protein sequence, changing how they tackle research questions and accelerate their projects. The methodology of AlphaFold and insights gained from the predictions for the complete human proteome have been described recently (9,13).

We present the AlphaFold Protein Structure Database (AlphaFold DB, https://alphafold.ebi.ac.uk), a new data resource created in partnership between DeepMind and the EMBL-European Bioinformatics Institute (EMBL-EBI). We have created AlphaFold DB to make structure predictions freely available to the scientific community at a large scale. The first release described here covers the human proteome and those of 20 other model organisms (Table 1). In the coming months, we plan to have expanded the database to cover a large proportion of all catalogued proteins (over 130 million cluster representatives from UniRef90).

**Table 1.** Structural predictions for complete proteomes in AlphaFold DB

Species	Common name	Reference proteome	Predicted structures
*Arabidopsis thaliana*	*Arabidopsis*	UP000006548	27 434
*Caenorhabditis elegans*	Nematode worm	UP000001940	19 694
*Candida albicans*	*C. albicans*	UP000000559	5974
*Danio rerio*	Zebrafish	UP000000437	24 664
*Dictyostelium discoideum*	*Dictyostelium*	UP000002195	12 622
*Drosophila melanogaster*	Fruit fly	UP000000803	13 458
*Escherichia coli*	*E. coli*	UP000000625	4363
*Glycine max*	Soybean	UP000008827	55 799
*Homo sapiens*	Human	UP000005640	23 391
*Leishmania infantum*	*L. infantum*	UP000008153	7924
*Methanocaldococcus jannaschii*	*M. jannaschii*	UP000000805	1773
*Mus musculus*	Mouse	UP000000589	21 615
*Mycobacterium tuberculosis*	*M. tuberculosis*	UP000001584	3988
*Oryza sativa*	Asian rice	UP000059680	43 649
*Plasmodium falciparum*	*P. falciparum*	UP000001450	5187
*Rattus norvegicus*	Rat	UP000002494	21 272
*Saccharomyces cerevisiae*	Budding yeast	UP000002311	6040
*Schizosaccharomyces pombe*	Fission yeast	UP000002485	5128
*Staphylococcus aureus*	*S. aureus*	UP000008816	2888
*Trypanosoma cruzi*	*T. cruzi*	UP000002296	19 036
*Zea mays*	Maize	UP000007305	39 299

AlphaFold DB provides free access to over 360,000 predicted structures across 21 proteomes. The data set contains proteins with sequence lengths of 16–2700 and excludes isoforms and sequences with unknown or non-standard amino acids.

## IMPLEMENTATION

The initial version of AlphaFold DB contains over 360 000 predicted structures, corresponding meta-information and confidence metrics. All the data are publicly accessible through a cloud-based infrastructure. We have attempted to predict most sequences in the UniProt reference proteome in the 16–2700 amino acid length range (as well as 1400-residue fragments to cover longer human proteins) for the organisms currently covered. We excluded sequences that contain non-standard amino acids. We do not provide multiple isoforms at this point.

The predicted structures contain atomic coordinates and per-residue confidence estimates on a scale from 0 to 100, with higher scores corresponding to higher confidence. This confidence measure is called pLDDT and corresponds to the model's predicted per-residue scores on the lDDT-Cα metric (14). lDDT is a pre-existing metric used in the protein structure prediction field. A key motivation behind lDDT is to assess the local accuracy of a prediction, awarding a high score for regions that are well-predicted even if the entire prediction cannot be aligned well to the true structure. This is particularly important for evaluating multi-domain predictions where the individual domains may be largely accurate while their relative position is not. As a confidence metric based on lDDT, pLDDT also reflects local confidence in the structure, and should be used, for example, to assess confidence within a single domain. Several other protein structure prediction resources also use lDDT-based metrics (15,16). AlphaFold DB stores these values in the B-factor fields of the mmCIF and PDB files available for download and uses confidence bands based on these values to colour-code the residues of the models in the 3D structure viewer on the structure pages. Residues with pLDDT ≥ 90 have very high model confidence, while residues with 90 > pLDDT ≥ 70 are classified as confident. Residues with 70 > pLDDT ≥ 50 have low confidence, and residues with pLDDT < 50 correspond to very low confidence (13). It was recently described that very low confidence pLDDT scores correlate with high propensities for intrinsic disorder (17).

The Predicted Aligned Error (PAE) is another output of the AlphaFold system. It indicates the expected positional error at residue *x* if the predicted and actual structures are aligned on residue *y* (using the Cα, N and C atoms). PAEs are measured in Ångströms and capped at 31.75 Å. Scientists can use these values to assess the confidence in the relative position and orientation of different parts of the model (e.g. two domains). For residues *x* and *y* in two different domains, if the PAE values (*x*, *y*) are low, AlphaFold predicts the domains to have well-defined relative positions and orientations. If the PAE values are high, then the relative position and orientation of the two domains are unreliable, and users should not attach biological or structural relevance to these. Note that the PAE is asymmetric; therefore, there can be a difference between the PAE values for (*x, y*) and (*y, x*), for example, between loop regions with highly uncertain orientation.

### Data archival

AlphaFold DB archives and provides access to the atomic coordinates in PDB and mmCIF formats, PAEs in JSON format and corresponding metadata in JSON format. While the coordinates and the PAE files are directly accessible through URLs, we load and index the metadata using the open-source search platform Apache Solr (https://solr.apache.org/) to enable users to search on the AlphaFold DB web pages. The data files in the archive are versioned, and previous snapshots of the data will be available via FTP, but the web pages will always display the latest version.

### Data access

AlphaFold DB provides predictions through several data-access mechanisms: (i) bulk downloads via FTP; (ii) programmatic access via an application programming interface (API); (iii) download and interactive visualization of individual predictions on protein-specific web pages keyed on UniProt accessions.

For bulk downloading data from AlphaFold DB, users can access the uncompressed archive files (.tar) of compressed PDB/mmCIF files (.gz) per reference proteome from the EMBL-EBI public FTP area at ftp://ftp.ebi.ac.uk/pub/databases/alphafold. This area contains the TAR files and a JSON file that provides meta-information, describing the species names (scientific and common), the reference proteome identifiers, the number of predicted structures, and the archives’ sizes. The same information and files are also available from the Bulk Download page of AlphaFold DB at https://alphafold.ebi.ac.uk/download.

We provide access to all entries through a public API endpoint, keyed on a UniProt accession. For example, the endpoint https://alphafold.ebi.ac.uk/api/prediction/Q92793 allows access to all the meta-information and the URLs of all the archived data files related to the human CREB-binding protein. UniProt (5), Pfam (18), InterPro (19) and PDBe-KB (7) use this API to display AlphaFold models on their web pages.

AlphaFold DB provides graphical access to and interactive visualization of all the predictions and meta-information for the broader scientific community through web pages. These pages contain all the available information for a protein of interest, keyed by its UniProt accession. They allow users to analyse the prediction and download the corresponding model files (in PDB and mmCIF formats) and PAE files (in JSON format).

### AlphaFold DB web pages

**Figure 1.**
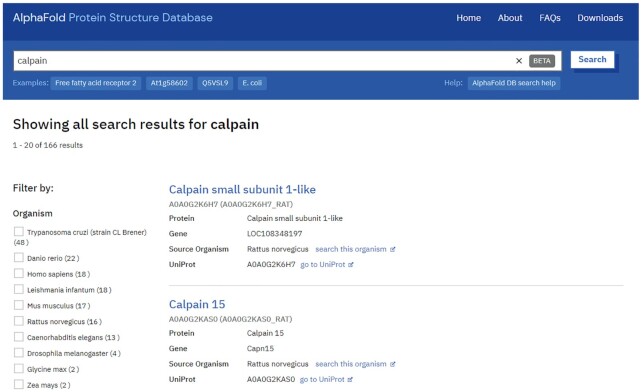
Searching AlphaFold DB. AlphaFold DB provides a search engine to find proteins of interest based on gene or protein name, UniProt accession or organism name. The search results can be filtered if required and clicking on a protein name leads to the relevant protein-specific entry page.

AlphaFold DB provides convenient access to its predictions through a set of web pages (https://alphafold.ebi.ac.uk). These pages contain an introduction to the AlphaFold system, address the most frequent questions, enable bulk download of complete proteomes, and offer a search engine for finding pages specific to a protein of interest (Figure 1). Users can search by gene name, protein name, UniProt accession or organism name. The search results can be filtered, for example, only to show human proteins.

**Figure 2.**
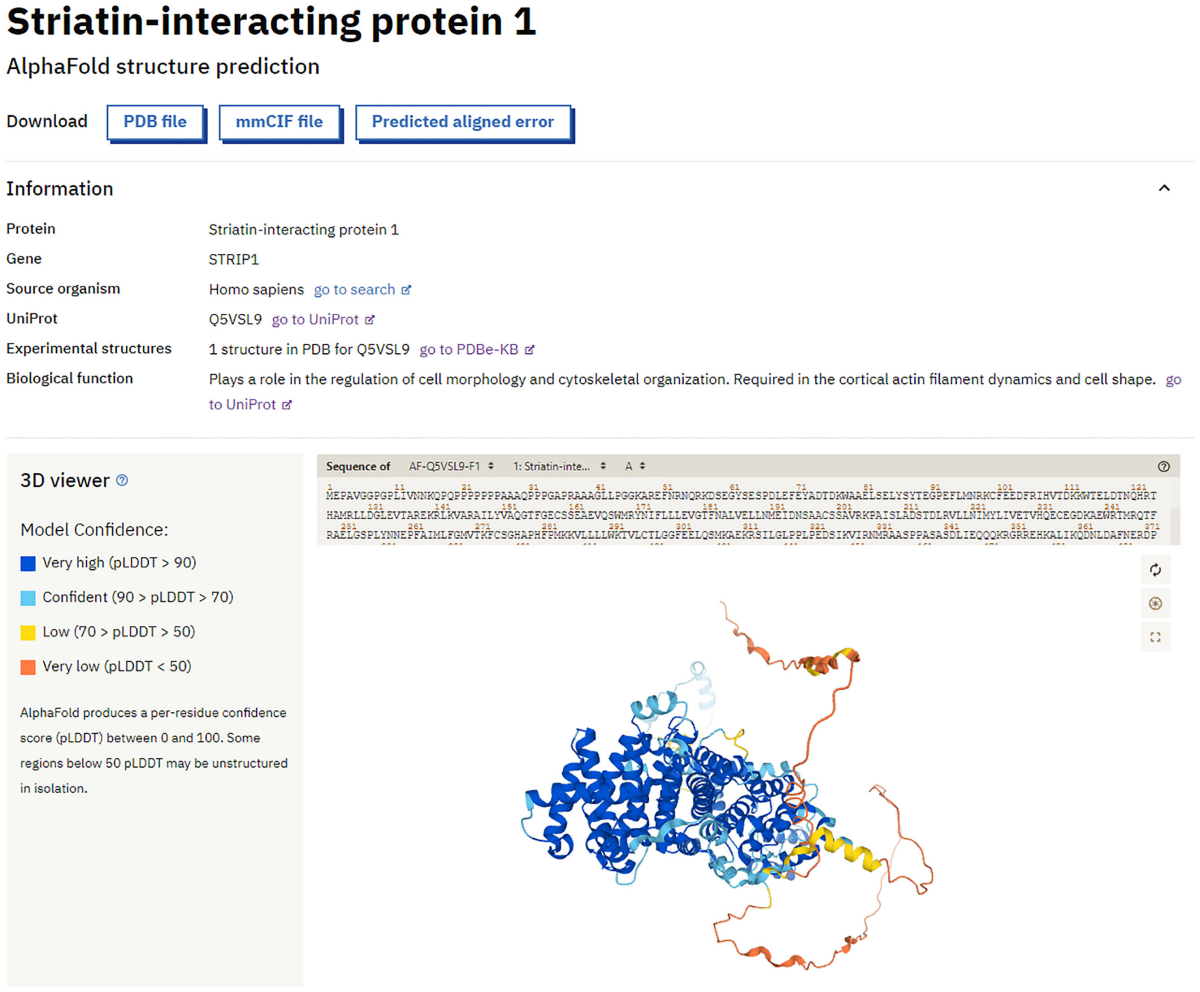
Meta-information and 3D visualization of the AlphaFold structure predictions. The protein-specific web pages display essential metadata for the protein of interest, such as known biological functions and cross-references to UniProt and PDBe-KB. Users can download the predicted models in PDB and mmCIF format, and an interactive molecular viewer visualizes the structure, coloured by the per-residue pLDDT confidence measure.

Each protein has a dedicated structure page that shows basic information (drawn from UniProt (5) and PDBe (6)) and three separate outputs of the AlphaFold model. The first two outputs are the 3D coordinates and the per-residue confidence metric pLDDT, which is used to colour the residues of the model in the integrated 3D molecular viewer, Mol* (20). Model confidence can vary significantly along a chain, making it essential to analyse the confidence measures before interpreting structural features. The lower confidence bands appear to correlate well with backbone flexibility and intrinsic disorder (13) (Figure 2).

**Figure 3.**
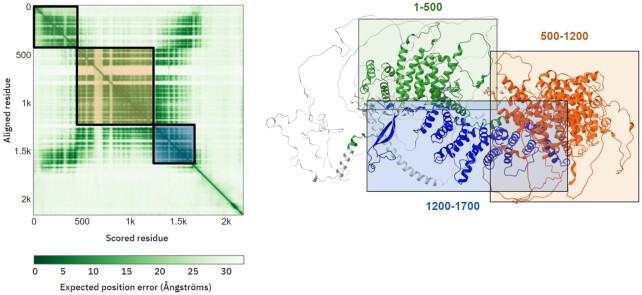
Visualization of Predicted Aligned Errors. Protein-specific pages contain an interactive 2D plot of the PAE values. This tool interacts with the 3D molecular viewer to facilitate the identification of domains whose relative positions and orientations AlphaFold predicts with confidence. In this example (https://alphafold.ebi.ac.uk/entry/Q93074), AlphaFold has high confidence in the relative position of domains at residues 1–500 (green) and residues 1200–1700 (blue), but not with the region between 500–1200 (orange) nor the C-terminus.

The third output is a pairwise confidence prediction, which helps to assess the reliability of relative domain positions and orientations as well as the global topology of the protein (Figure 3). The plot is coloured by the pairwise PAE values and it helps users to identify which domains have reliably predicted positions and orientations relative to one another, where dark green indicates high confidence. Selecting a region in the plot also highlights the corresponding part of the sequence in the 3D viewer.

## CONCLUSION AND OUTLOOK

Since the mid-1950s, the scientific community has been using ever-more advanced experimental methods to determine over 180 000 structures of proteins, nucleic acids, and complexes in atomic detail, and archive them in the PDB, the single worldwide archive of experimental macromolecular structure data managed by the wwPDB consortium (21). This collective body of work has vastly improved our understanding of many fundamental processes in health and disease, as evidenced in part by many Nobel Prizes for structures deposited in the PDB. Recently, determining the structure of the SARS-CoV-2 viral proteins enabled scientists to understand how it operates and to identify potential treatments and develop new vaccines (3). However, figuring out the exact structure of a protein remains an expensive and often time-consuming process. Thus, we only know the 3D structure of a tiny fraction of all proteins currently known to science.

The first release of AlphaFold DB contains over 360 000 predicted structures from 21 model-organism proteomes. Having access to these highly accurate models will greatly impact biology, from enabling structure-based drug design to providing data for high-throughput structural bioinformatics research that will tackle fundamental biological questions. We have already gained some invaluable insights from the predictions of the human proteome (13).

In the coming months, we will expand AlphaFold DB to provide structural predictions to include additional proteomes to support research in neglected diseases and to cover the set of highly annotated proteins in SwissProt, taking the number of structures available to >1 million. This will be followed by another update in 2022 to include structures for most representative sequences from the UniRef90 data set (>100 million structures). Future updates will also aim to overlay annotations onto the predicted structures and display this information on 2D sequence-feature viewers. AlphaFold DB will enable biomedical scientists to use 3D models of protein structures as a core tool, driving research and innovation across multiple fields by providing open access to an ever-growing number of predicted structures.

## DATA AVAILABILITY

All the AlphaFold predictions are publicly available through multiple data-access mechanisms. Coordinate files in PDB and mmCIF formats are available in TAR archives per proteome through FTP at ftp://ftp.ebi.ac.uk/pub/databases/alphafold. Meta-information and URLs to individual UniProt accessions are available via a public API endpoint. For example, https://alphafold.ebi.ac.uk/api/prediction/Q92793 provides all the information for UniProt accession Q92793 (https://www.alphafold.ebi.ac.uk/entry/Q92793).
